# Innate Lymphoid Cells - Neglected Players in Multiple Sclerosis

**DOI:** 10.3389/fimmu.2022.909275

**Published:** 2022-06-17

**Authors:** Negar Sadeghi Hassanabadi, Bieke Broux, Sonja Marinović, Dagmar Gotthardt

**Affiliations:** ^1^ Institute of Pharmacology and Toxicology, University of Veterinary Medicine Vienna, Vienna, Austria; ^2^ University MSCenter; Campus Diepenbeek, Diepenbeek, Belgium; ^3^ Neuro-Immune Connections and Repair Lab, Department of Immunology and Infection, Biomedical Research Institute, UHasselt, Diepenbeek, Belgium; ^4^ Division of Molecular Medicine, Laboratory of Personalized Medicine, Ruder Boskovic Institute, Zagreb, Croatia

**Keywords:** multiple sclerosis, innate lymphoid cells (ILCs), natural killer cells, experimental autoimmune encephalomyelitis (EAE), autoimmune disease, disease-modifying therapies (DMTs)

## Abstract

Multiple sclerosis (MS) is a highly debilitating autoimmune disease affecting millions of individuals worldwide. Although classically viewed as T-cell mediated disease, the role of innate lymphoid cells (ILC) such as natural killer (NK) cells and ILC 1-3s has become a focal point as several findings implicate them in the disease pathology. The role of ILCs in MS is still not completely understood as controversial findings have been reported assigning them either a protective or disease-accelerating role. Recent findings in experimental autoimmune encephalomyelitis (EAE) suggest that ILCs infiltrate the central nervous system (CNS), mediate inflammation, and have a disease exacerbating role by influencing the recruitment of autoreactive T-cells. Elucidating the detailed role of ILCs and altered signaling pathways in MS is essential for a more complete picture of the disease pathology and novel therapeutic targets. We here review the current knowledge about ILCs in the development and progression of MS and preclinical models of MS and discuss their potential for therapeutic applications.

## Introduction

Multiple sclerosis (MS) is a chronic autoimmune, inflammatory neurological disease of the central nervous system (CNS) that most commonly affects young individuals between the age of 20 and 40 (1). The number of people worldwide living with MS has increased from 2.3 million in 2013 to 2.8 million in 2020 becoming the primary cause of non-traumatic disability in young adults (1). Although disease-modifying therapies (DMTs) have alleviated symptoms and reduced the subsequent disability coming from an MS diagnosis, the disease progression still cannot be stopped (2). This is due to a complex disease etiology and pathogenesis, highlighting the unmet need to identify key players and altered drivers in MS. For decades, MS has been viewed as an immune-mediated disease primarily induced by the infiltration of classical T-cells (2). Subsequently, studies on MS and experimental autoimmune encephalomyelitis (EAE) have mostly focused on CD4^+^ T-cells, and DMTs that have been developed over the years mainly target these cells. The traditional view has been altered to include the involvement of innate immune cells that have long been neglected as disease mediators. Most studies investigating the role of ILCs in MS and preclinical models of MS have been focused on NK cells as ILC1-3 have been identified and classified later. Nonetheless, recent studies suggest that also other ILC family members play a crucial role in the initial activation of the autoimmune response as well as the disease progression, making them a potential target for future therapies (3–19).

## Multiple Sclerosis

MS can be divided into 3 subtypes: relapse remitting MS (RRMS), primary progressive MS (PPMS), and secondary progressive MS (SPMS) (20). The most common type is RRMS, which is characterized by recurring episodes of neurological dysfunction, followed by a clinical recovery. Many RRMS patients develop SPMS within 10 to 15 years after the RRMS diagnosis, whereby inflammatory lesions are no longer the main characteristic. Instead, progressive neurological decline occurs, followed by brain atrophy (2). Only 10 to 15% of the patients have PPMS, whereby disability progression is present from the beginning of the disease and relapses do not occur (20). The disease symptoms are heterogeneous and depending on where the lesions are in the CNS, sensory disturbances, bladder dysfunction, cognitive deficits, limb weakness, ataxia, and fatigue can occur (21). The disease pathology of MS is characterized by confluent demyelinated areas in both the white and gray matter of the brain and spinal cord. These lesions indicate loss of myelin and myelin-producing oligodendrocytes, resulting in disrupted conduction of electrical impulses (2). The pathology is due to an autoimmune response directed against myelin, whereby immune cells such as CD4^+^ T-cells and B-cells infiltrate the brain parenchyma and cause local tissue damage. In addition, ectopic lymphoid follicles (ELFs), which resemble germinal center-like structures can be found in the meninges of 40% of the SPMS patients. ELFs were also observed in RRMS and PPMS patients but lack features of more developed follicles such as follicular dendritic cells (FDCs), and distinct T and B cell zones (22, 23). ELFs can cause local antigen-specific responses within tissues and thereby support cortical degeneration and clinical disease progression (22). Although the causes of MS are still unknown, next to a genetic predisposition, many different environmental factors such as vitamin D deficiency, obesity, smoking, and infection with the Epstein Barr virus (EBV) have been described to play a role in developing MS (2). Emerging evidence from a longitudinal study shows that an EBV infection increases the risk of getting MS 32-fold, suggesting that EBV is the leading cause of MS (24).

## Innate Lymphoid Cells

**Figure 1:**
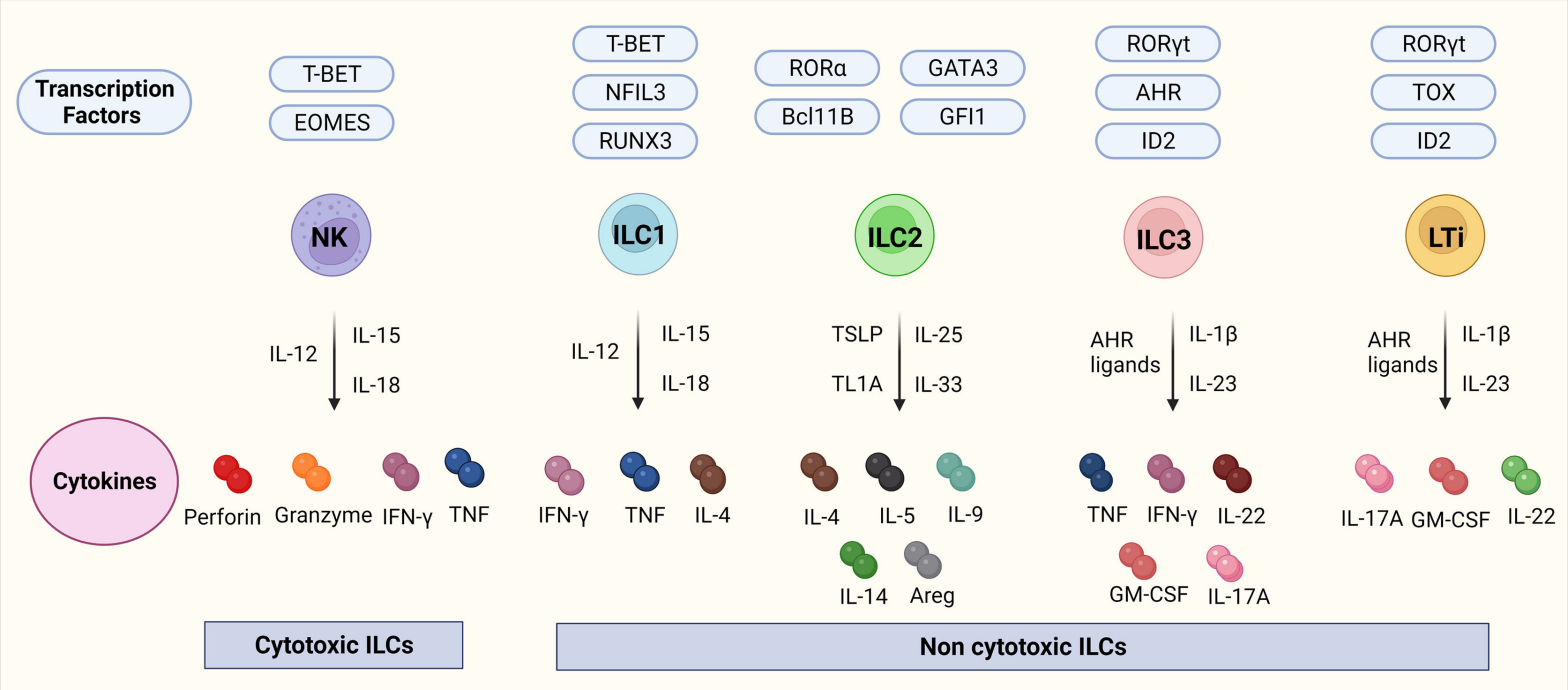
ILCs, their transcription factors, and cytokines. **Figure 1** depicts the ILC family, their transcription factors, and cytokines in mice. NK cells are transcription factor T-bet and Eomes dependent cytotoxic ILCs that release cytokines IFN-γ, and TNF together with cytotoxic molecules such as perforin, and granzyme. ILC1-3 and LTis are non-cytotoxic ILCs. ILC1s are dependent on the transcription factors Tbet, NFIL3, and RUNX2 and release IFN-γ, TNF, and IL-4. ILC2s are dependent on the transcription factors RORα, GATA3, Bcl11B, and GFI and release IL-4, IL-5, IL-9, IL-14, and transcription factor Areg. ILC3s are dependent on the transcription factors RORγt, AHR, and ID2 and release TNF, IFN-γ, IL-22, GM-CSF, and IL-17A. LTis are dependent on the transcription factors RORγt, TOX, and ID2 and release IL-17A, GM-CSF, and IL-22. Abbreviations of transcription factors: NFIL3, nuclear factor IL-3 induced; ID2, inhibitor of DNA binding 2; TOX, thymocyte selection associated high mobility group box protein; GATA3, GATA binding protein 3; T-BET, T-box transcription factor; EOMES, Eomesodermin; RUNX3, runt-related transcription factor 3; RORα, RAR-related orphan recepto;, Bcl11b, B cell lymphoma/leukemia 11B; RORγt, RAR- related orphan receptor γt; and AhR, Aryl hydrocarbon receptor. Abbreviations of cytokines: IFN-γ, Interferon-gamma; TNF, Tumor necrosis factor-alpha; IL, Interleukin; GM-CSF, Granulocyte-macrophage colony-stimulating-factor; Areg, amphiregulin.

ILCs are a branch of the innate immune system and an important source of innate effector cytokines (25, 26). In the last years, their resemblance to T-cells has been described, which lead to the recognition of ILCs as the innate counterparts of T-cells whereby each subset resembles a specific T-cell population in particular. There are different nomenclatures regarding ILCs, but in this review, we use the nomenclature proposed by Vivier et al. (27). ILCs can be divided into five different subsets: lymphoid tissue inducers (LTis); cytotoxic NK cells, which enter the circulation and migrate through tissues; and ILC1, 2, and 3 which are tissue-resident non-cytotoxic cells that exert their effects locally (25, 28). Interestingly, recent studies have identified ILC1 and ILC3 subsets that also have a cytotoxic nature (25, 28) suggesting that some key features are yet to be discovered in terms of subsets, functions as well as plasticity. LTis, NK cells, and ILC1, 2, and 3 differ in their transcription factor profiles as well as functions. NK cells resemble CD8^+^ cytotoxic T-cells whereas ILC1-3s, mirror CD4^+^ T helper (Th)1, Th2, and Th17 in terms of function (25, 26) which is visualized in **Figure 1**.

### NK Cells

NK cells are dependent on the transcription factors, T-bet (Tbx21) and Eomesodermin (Eomes) and are cytotoxic cells that control tumors and viral infections by producing IFN-γ, TNF, perforin, and granzymes (25). In addition, NK cells exert regulatory effects by influencing adaptive immune responses (27). In mice, three NK cell populations have been characterized, based on their CD11b and CD27 expression (29). The double-positive and the most mature subset CD11b^+^CD27^+^ have cytolytic potential and secrete IFN-γ being the most potent killer cell population while CD11b^+^ CD27^-^ cells have lower proliferative capacity indicating replicative senescent cells (29, 30). The NK cells subsets not only differ in functions but as well in their tissue distribution; the CD11b^-^ CD27^+^ NK cells are mostly found in the bone marrow and lymph nodes, whereas the CD11b^+^ CD27^-^ NK cells are predominantly found in the blood, spleen, lung, and liver. The double-positive NK cells can be found in all tissues and are evenly distributed (29). In humans, NK cell maturation can be distinguished based on CD56 and CD16 expression – functional homolog to the CD27/CD11b subsets in the mouse. Human NK cells are mainly divided into immature CD56^bright^CD16^-^ and mature CD56^dim^CD16^+^ and subsets (31). In the peripheral blood and spleen, around 90% of the NK cells are CD56^dim^CD16^+^. These cytotoxic cells express perforin and *in vitro*, it was shown that they can produce IFN-γ upon interaction with tumor cells (32). In the lymph nodes and tonsils, most NK cells are CD56^bright^CD16^-^ and lack perforin but produce cytokines such as IFN-γ in response to stimulation with interleukin (IL)-12, 15, and 18 (31, 33). Although there are differences between human and mouse NK cells such as the absence of the NKp30 and NKp44 activating receptors and the CD56 marker in mice, many fundamental principles such as the biology and function of NK cells can be studied in mice and applied to humans (34).

### ILC1

ILC1s defend against intracellular bacteria and parasites and play a role against tumor and virus-infected cells by producing cytokines (25). ILC1s, although having similar features as NK cells such as the production of IFN-γ, can be distinguished from conventional NK cells as they are only dependent on the transcription factor T-bet but not Eomes. Their similarity to NK cells causes a hurdle to correctly identify them, as specific ILC1 markers are lost upon cell activation and are tissue-dependent. As well, many ILC1 markers are shared with NK cells and ILC3s such as NK1.1, NKp44, and NKp46 (26). ILC1s can be further divided into CD127^-^ and CD127^+^ cells whereby CD127^-^ cells can produce IFN-γ and TNF upon stimulation by IL-12, IL-15, and IL-18, and CD127^+^ cells produce IFN-γ in response to IL-12 and IL-18 in both mouse and humans (25).

### ILC2

ILC2s are important for the defense against parasites and are involved in asthma and allergic diseases (24). They are dependent on the GATA3 transcription factor and when activated *via* cytokine receptors, produce type 2 cytokines such as IL-4, IL-5, IL-9, and IL13, and the transcription factor Areg in both mice and humans (24). ILC2 can be identified by ST2, a component of the IL-33 receptor (25).

### ILC3s

ILC3s are dependent on the transcription factors RORγt, AHR, and ID2. They are significant producers of IL-17A, IL-22 as well as GM-CSF and TNF and promote antibacterial immunity, chronic inflammation, and tissue repair as well they can regulate adaptive Th17 cell response (25). ILC3s are heterogeneous and in mice, there are two subsets, CCR6-ILC3s whereas in humans all express CCR6 and CD117 but two subsets can be distinguished based on NKp44 expression (25).

### LTis

LTis are CCR6^+^ transcription factor RORγt - dependent cells and during embryonic development are crucial for the formation of secondary lymph nodes and Peyer’s patches (PP) (35). In mice, there are two different subsets, which can be differentiated based on their CD4 expression. They produce similar cytokines as ILC3 whereby the only difference is that they produce IL-17A instead of TNF (35).

## Role of ILCs in MS

### EAE – A Preclinical Model for MS

The experimental autoimmune encephalomyelitis (EAE) model is the most commonly used mouse model to study the immune response in MS. EAE is a CD4^+^ T cell-driven disease, directed against myelin antigens (36). Since it was first introduced, over 60 years ago, it has been extensively used to understand the autoimmune contribution to the pathogenesis of MS (37). EAE is induced by active immunization with myelin proteins or peptides derived from the myelin sheath, whereby the most common models are myelin oligodendrocyte glycoprotein (MOG_33-55_) immunized C57BL/6 mice or PLP_139-151_ SJL mice. The mice present symptoms such as loss of appetite, ascending paralysis, and significant inflammatory infiltration which can be found predominantly in the spinal cord but also the brain and which worsens with time (36). The MOG_33-55_ immunized C57BL/6 mice and PLP_139-151_ immunized SJL mice mimic different disease stages. The SJL mice show similarities to a relapse–remitting disease course, which is the most common form of MS. Furthermore, it also mimics a sex-dysmorphism that can be found in humans (38). The MOG_33-55_ immunized C57BL/6 mice are more useful to mimic the chronic stages of MS (37). Although differences between the pathophysiology of the EAE animal model and human MS exist, EAE is a very powerful tool for understanding the autoimmune and inflammatory parts of the disease. In addition, it is essential for the development of therapies and is used in pre-clinical trials to test the efficacy of drug candidates (37). Indeed, the use of EAE in pre-clinical trials has been essential for MS drugs such as Interferon beta IFN-β, Glatiramer acetate (GA), and Natalizumab (39).

### NK Cells in EAE

In the last decade, the role of NK cells in MS has been investigated and both the beneficial and harmful role of NK cells has been demonstrated (40, 41). The fact that NK cells could play a role in MS, arises from findings of studies in MS patients, the EAE model, and DMTs influencing NK cell numbers as well as function and migration (3, 4, 40) (**Table 1**).

**Table 1:** Overview of ILCs in preclinical models of MS and in MS patients.

ILC type	Experiment	Outcome	Role of ILC
**NK cells**	NK1.1 depleted C57BL/6 mice EAE induced with MOG_35-55_ (40)	Enhanced disease progression	Protective
	NK1.1 depleted 2m^-/-^ mice EAE induced with MOG_35-55_ (40)	Enhanced disease progression	Protective
	NK1.1 depleted 2m^-/-^ mice EAE induced with MOG_35-55_ (40)	Enhanced disease progression	Protective
	NK.1.1 depleted SJL/J mice one day before and 14 days after EAE induction with PLP_136–150_ (5)	Enhanced disease progression	Protective
	NK1.1,/-asialo GM1/Ly49 depleted C57BL/6 mice one day before and before secondary EAE induction with MOG_35-55_ (41)	Diminished EAE onset	Pathogenic
	NK1.1 depleted IL-18^-/-^ mice EAE induced with MOG_35-55_ (6)	Resistant to EAE	Pathogenic
	Eomes ^f/f^ NKp46-Cre^+^ mice EAE induced with passive transfer of 2D2 wild-type Th17 cells (4)	Equal disease progression	None
**ILC1**	Tbx21^-/-^ mice EAE induced with MOG_35-55_ (4)	Diminished EAE onset/progression	Pathogenic
	NK1.1 depleted Tbx21^−/−^ mice EAE induced by adoptive transfer of autoreactive CD4^+^ Th17 (4)	Resistant to EAE	Pathogenic
	Tbx21^f/f^ NKp46-Cre^+^ EAE induced with MOG_35-55_ (4)	Diminished EAE onset	Pathogenic
	ILC1^-/-^ HSV-IL2 mice (16)	Demyelination comparable to control mice	None
**ILC2**	ILC2^-/-^ HSV-IL-2 mice (16)	Protection from demyelination	Pathogenic
	ILC2^-/-^ with adoptive BM-derived ILC2s and infection with HSV-IL-2 virus (16)	Severe demyelination in comparison to control mice	Pathogenic
	SJL-cKit^-/-^ mice EAE induced with PLP_136–150_ (17)	c-Kit mutation induces severe EAE in males but not females	Protective
**ILC3**	Thy1^+^ depleted mice EAE induced with MOG_35-55_ (42)	Equal disease progression	None
	C57BL/6 mice EAE induced with MOG_35-55_ (42)	Increased number of IL-17, TNF and IFN-γ producing ILC3s in the meninges	Pathogenic?
	ILC3^-/-^ HSV-IL2 mice (16)	Demyelination comparable to control mice	None
**LTis**	MS patients (43–46)	Elevated levels in the CNS and blood	Pathogenic?
	CSF samples from MS patients (43)	Increased frequency of CD56^-^RORyt^+^ LTis	Pathogenic?
	Rorc^-/-^ mice EAE induced with MOG_35-55_ (42)	Resistant to EAE	Pathogenic

A study done in 1997, showed that C57BL/6 mice deprived of NK cells, using an anti-NK1.1 depletion antibody before EAE induction with MOG_35-55_, resulted in a serious form of EAE with frequent relapses which occurred earlier than in EAE induced control mice (40). To test that the seen effects were not caused by the depletion of NK-T cells, which are also targeted by an anti-NK1.1 antibody, they used a β2-microglobulin^-/-^ β2m-/- mice in which NK-T-cells are absent. The β2m-/- mice depleted from NK cells developed a chronic non-remitting form of EAE with high clinical scores, providing evidence that NK cells can play a regulatory role in a manner independent of CD8^+^ T cells or NK1.1^+^ T cells. In addition, in the same study enhanced disease progression was observed upon NK cell depletion in Rag2*^-/-^* mice lacking T-, NK-T, and B-cells after disease induction by adoptive transfer of MOG-specific T-cells (40). This study clearly indicates a disease protective role of NK cells (40). Similar results were found in NK-depleted SJL/J mice (5), whereby the depletion of NK cells by an anti-NK1.1 monoclonal antibody, one day before and 14 days after the immunization with PLP_136–150_ lead to an enhanced form of EAE (5). The authors describe that the disease protection of NK cells was mediated by a cytotoxic effect on autoantigen-specific encephalitogenic T-cells, which are known to play a vital role in the autoimmune attack in MS (5). In contrast to this, another study demonstrated an NK cell depletion with either anti-NK1.1, anti-asialo GM1, and anti-Ly49 in C57BL/6 before EAE induction with MOG_35-55_ results in a decrease in clinical pathology and relapses (41). The authors also report higher survival, thereby showing a disease-accelerating role of NK cells in EAE (41). It must be noted that an NK1.1 antibody depletes not only NK cells but also ILC1s and partially ILC3s and does not allow for discrimination between the role of these cell types whereas the used anti-asialo GM1 and anti-Ly49H preferentially deplete NK cells. The authors also state without providing the data that depletion of NK cells after the immunization did not alter the clinical symptoms in the mice which supports the notion that NK cells have only a regulatory role early in the disease development. Another study supported the disease-accelerating role of NK cells which was based on the fact that IL-18 can promote the production of IFN-γ by NK and Th1 cells. In the study, IL-18^-/-^ mice were completely protected from EAE after MOG immunization, while IL-18 administration restored the disease partially in the presence of NK cells but not when NK1.1^+^ cells were depleted before immunization (6). Likewise as mentioned above the study also notes that NK depletion after primary immunization has no effects on the EAE course further providing evidence that NK cells have a role in the initiation of the disease but not in the progression.

### NK Cells in MS

Human studies have implicated NK cells directly in the process of demyelination and thus support the notion of a disease-accelerating role. In a study, using human brain-derived oligodendrocytes (OLs) from surgery, it was found that CD3^+^CD19^+^CD14^+^ depleted mononuclear cell preparations (MNCs) were cytotoxic toward OLs (7). This effect was visible when using NK cell-enriched MNCs and OLs from either the same donor (autologous donors) or healthy volunteers and OLs from epilepsy patients (heterogenous donors). NK cell-mediated cytotoxicity towards OLs was further increased when NK cells were activated with IL-2. The same group showed in another study that OLs from patients with MS lesions express MICA/B ligands for the activating NK cell receptor NKG2D that were not detected in healthy control samples. Blocking NKG2D on NK cells significantly inhibited the killing of OLs indicating an NKG2D-mediated killing of OLs by NK cells (8). Using cortical CNS biopsies of patients diagnosed with inflammatory demyelination a study showed the presence of brain perivascular granzyme B^+^ NK cells. The authors further showed in a Th/^+^ (BCR transgenic for MOG) mouse model that NK cells strongly aggravate the extent of perivascular cortical demyelination, providing evidence for the relevance of NK cells in perivascular cortical demyelination and contribution to the neurodegeneration of MS (9). In addition, autoreactive 2D2 T-cells were either transferred into RAG1^−/−^ (lacking mature T– and B–cells) or into RAG1^−/−^ γc^−/−^ (no mature T– and B–cells, no NK cells) mice and the latter ones showed significantly less perivascular cortical demyelination. However, the extravasation of NK cells into the cortical parenchyma required activated T cells. These data suggest that NK cells contribute to the demyelination process in the presence of pathogenic antibodies by performing ADCC (10).

Recently, EBV infection has been proven to be a leading cause of MS (24). As NK cells protect against viral infections, they play a vital role in the early defense against an EBV infection (11). Findings suggest that during infectious mononucleosis (IM) caused by a primary EBV infection, NK cell numbers were significantly elevated both at the diagnosis and during the first month of an IM diagnosis (12, 13). Furthermore, significant changes in cell phenotype and function of NK cells were also detected.

### ILC1

Induction of EAE in transcription factor T-bet-deficient mice showed that myelin-reactive pathogenic CD4^+^ Th-17 cells invaded the CNS and caused major lesions throughout the CNS tissue in immuno-competent hosts but were completely absent from the CNS parenchyma in T-bet-deficient hosts (4). Furthermore, disease induction by an adoptive transfer of autoreactive CD4^+^ Th17 cells into NK1.1 depleted mice resulted in resistance to EAE. In addition, mice with NKp46-lineage-specific deletion of the transcription factor T-bet significantly decreased the incidence of inflammation using the adoptive transfer model (4). Interestingly, the study also used NKp46 lineage-specific deletion of transcription factor Eomes in mice as a tool to study the absence of conventional NK cells only and found that these NK cell-deficient mice developed a similarly severe paralysis after EAE induction compared to NK cell-sufficient mice. This indicates, in contrast to prior studies, that ILC1s but not NK cells play a role in the immunopathogenesis of EAE. The authors concluded that the pathogenic function of the transcription factor T-bet is rather dependent on ILC1 and the NKp46^+^ subset of ILC3s. A study investigating phenotypic and functional plasticity of murine ILCs found that the meningeal subsets of NKp46^+^ ILC1s and ILC3s are also characterized by a specific gene signature that was not found in splenic or meningeal NK cells and might contribute to the disease pathology (14). Another study found an increase of ILC1s in the meninges of EAE-diseased in comparison to healthy wild-type C57BL/6 mice (19). Others also found that in contrast to NK cells, ILC1s are enriched in the choroid plexus as well as in the brain parenchyma and meninges after EAE induction (15). However, while ILC1s maintain stable IFN-γ and TNF levels, NK cells show increased production of these cytokines during EAE progression. These findings at least indicate again that besides NK cells, CNS-ILC1s could be involved in the control of neuroinflammation in the brain (15).

### ILC2

ILC2s are the predominant ILC population in human as well as mouse brains and have been implicated in the development of several diseases such as allergy, asthma, dermatitis, and fibrosis (16). Studies also suggest the involvement of ILC2s in the disease pathology of MS (15, 16, 40). Like ILC1s, ILC2s have also been found in the meninges of wildtype C57BL/6 mice (19). A study from 2020, using an HSV-IL-2 model of CNS demyelination where mice were infected with a recombinant HSV-1 expressing murine interleukin-2 showed that NK cells did not play a role in demyelination in this model (16). However, ILC2^-/-^ mice infected with the HSV-IL-2 virus were protected from demyelination whereas ILC1^-/-^ and ILC3^-/-^ mice showed demyelination to the same extent as wild type mice. Additionally, adoptive transfer of bone marrow-derived ILC2 from wild-type mice into ILC2^-/-^ infected HSV-IL-2 virus restored demyelination visible in the brain, spinal cord, and optic nerve implying that CNS demyelination is dependent on ILC2s but not ILC1s and ILC3s (16). As previously mentioned, studies of EAE in SJL mice serve as a useful model of sex-dimorphism in MS (38). A recent study suggested that the female bias for disease development comes from a lack of male-specific IL-33 expression in mast cells, likely influenced directly by testosterone (17). Interestingly, instead of IL-33, immunized wild-type females express IL-1β and TNF that exert a variety of pathogenic effects and disease promotion. IL-33 plays an important role in the activation of ILC2s, a population that is essential for skewing the immune environment towards a protective Th2-dominated response of wild-type males rather than the harmful Th17-dominated response seen in females (17). The protective role of ILC2s was further corroborated in SJL-cKit mutant mice (SJL-Kit^W/Wv^) where it was shown that c-kit mutations induce severe EAE in males, but not females, due to the deficit of c-kit^+^ ILC2s and mast cells (18).

### ILC3

A study investigating the contribution of ILCs to autoimmune neuroinflammation *via* their immediate responsiveness to IL-23 signaling found that ILC3s are not only present at mucosal surfaces but indeed can be found in the CNS during steady-state and inflammation (19). Especially IL-17A-producing ILC3s have been found in the CNS but the depletion of Thy1^+^ ILCs did not alter the progression of EAE (19). The same study that identified ILC1s and ILC2s in meninges of wildtype C57BL/6 mice identified an ILC3 subset (CD45^+^Lin^-^IL-7Ra^+^RORγt^+^), which also resides in the meninges (42). To assess the role of this ILC3 subtype, the authors induced EAE with MOG_35-55_ in the C57BL/6 mice and found an increased number of IFN-γ, GM-CSF, and IL-17 producing ILC3s in the meninges of EAE induced mice. It is therefore assumed that this subset plays an essential role in MS by promoting inflammation and thereby creating an environment that promotes the survival and reactivation of encephalitogenic T-cells (42).

### LTis

LTi cells are elevated in the CNS and circulation of MS patients, providing evidence for a possible pathogenic role of LTis (42–45). A study published in 2016, used Cerebrospinal fluid (CSF) samples from MS patients and healthy controls to compare the levels of different ILCs (43). They found that CSF samples from MS patients compared to healthy controls have an increased frequency of CD56^-^ RORγt^+^ LTis while the frequency of CD56^+^ RORγt ILC3s was unaltered. LTis are important producers of IL-22 and during an MS relapse, the balance of cytokines is shifted towards a pro-inflammatory profile whereby IL-22 is assumed to promote blood-brain barrier (BBB) disruption and CNS inflammation (42). The same study that found an increased number of ILC3s in the meninges of EAE induced mice, showed that also LTis are significantly increased in the meninges and the CNS at the peak of EAE (19). They also showed that Rorc^-/-^ mice are protected against EAE, which might be due to the lack of LTis or their subsequent inability to mobilize to the meninges providing further support for the involvement of LTis in the development of ELFs (42). That LTi subtypes also reside in the meninges, is an important finding as LTis are also speculated to be involved in the development of ELFs in MS patients, and ELFs are commonly formed in the meningeal tissues in humans and mice. It is assumed that the LTi subset residing in the meninges has a direct role in orchestrating the formation of ELFs, which are hallmarks of chronic autoimmune inflammatory diseases such as MS.

**Figure 2:**
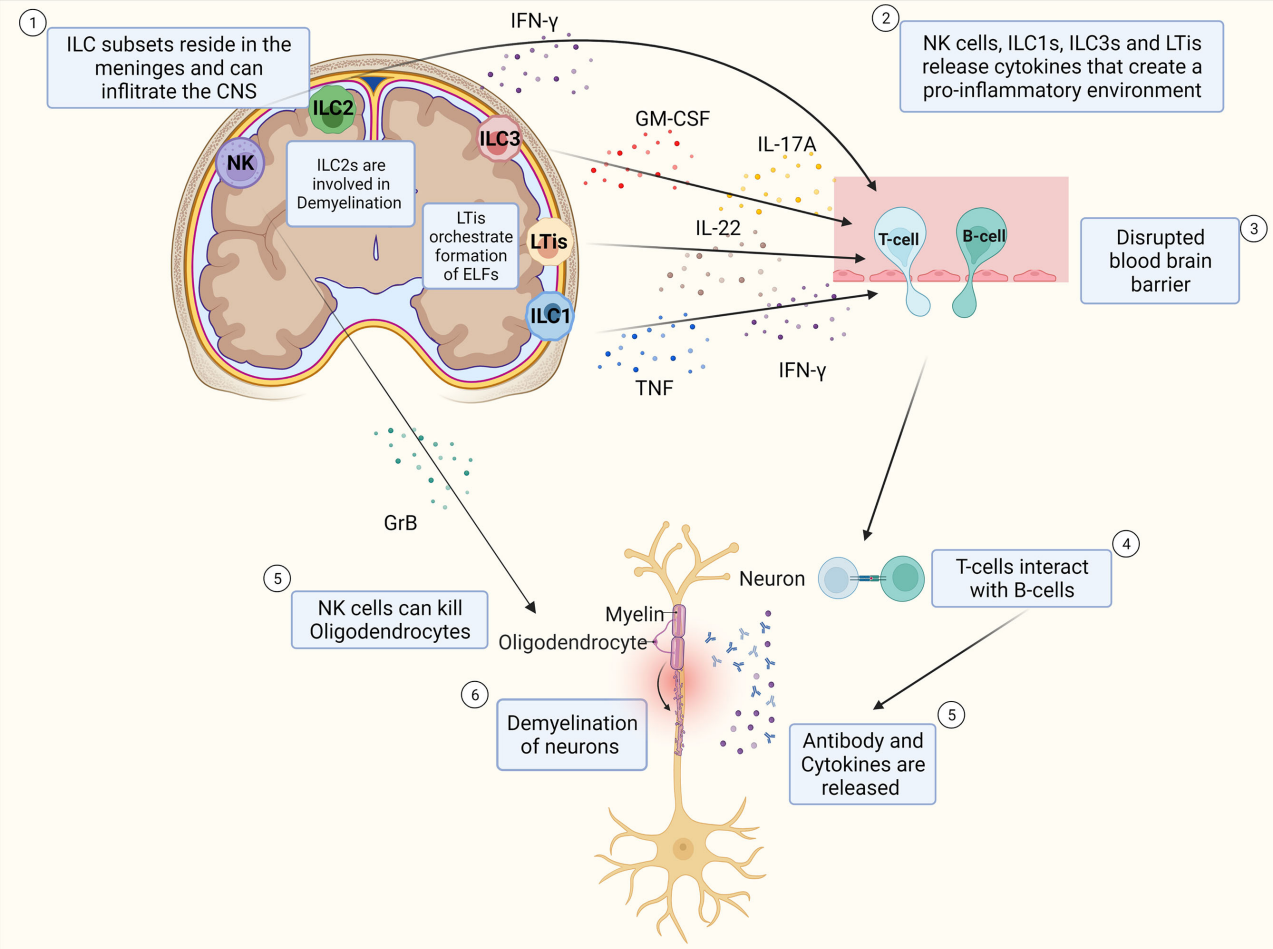
Schematic Overview of potential pathophysiology of MS and role of ILCs. T-cells and B-cells infiltrate the CNS through a leaky blood-brain barrier whereby ILC subtypes that reside in the meninges release pro-inflammatory cytokines that influence the extent of CD4^+^ T-cell infiltration into the CNS. T-cells interact with B-cells and release cytokines and antibodies that cause inflammation which damages the myelin sheath of neurons and thereby induce demyelination. NK cells can also kill oligodendrocytes directly thereby potentially contributing to the extent of demyelination of the neurons. ILC2s are assumed to play a role in demyelination. LTis are assumed to play a role in the development of ELFs.

Together, these findings provide evidence that ILC subtypes reside in the meninges and can infiltrate the CNS. Next to this, these findings show that ILCs release cytokines that regulate the inflammatory response of T-cells, as well as cytokines that are pro-inflammatory and exacerbate the immune response in MS, which can be seen in **Figure 2**. The specific role of ILCs is still unclear as contradictory results have been found.

### Role of ILCs in MS: Lessons Learned From DMTs

Next to IFN-β and Glatiramer acetate other immunomodulatory therapies for MS have been approved in the last years, such as the anti-inflammatory Dimethyl fumarate (DMF), the highly potent migration inhibitors such as Natalizumab and Fingolimod (FTY720), and the IL-2 receptor modulating Daclizumab (10). Importantly, all aforementioned drugs also influence NK cells next to their original target. Some drugs, such as Daclizumab and Fingolimod, show an additional effect on other members of the ILC family (**Table 2**).

**Table 2:** Overview of DMTs for MS influencing ILCs.

Drug	Mechanism	ILC type	Effects on ILCs
Interferon beta 1a	Reduction of T-cell proliferation (47) Reduction of MHC class II molecule expression (47) Lowered IFN-β production by Th17 cells (47)	NK cells	Upregulation of MHC class I dampened cytotoxicity of CD56^dim^ NK cells (47) Altered CD56^dim^/CD56^bright^ population ratio with an expansion of CD56^bright^ NK cells (48, 49)
Natalizumab	Humanized alpha 4 integrin antibody inhibiting leukocyte migration (50)	NK cells	Increase of total NK cell numbers (including CD56^bright^ NK cells) in the blood and reduced NK cell numbers in the CSF (50, 51)
**Daclizumab	Monoclonal antibody against IL2 receptor CD25 (52)	NK cells ILCs	Expansion of immunoregulatory CD56^bright^ NK cells in peripheral blood and CSF (51, 53) Decrease in circulating ILCs and shift in LTi phenotype towards NK lineage (43)
Alemtuzumab	Monoclonal antibody specific for the membrane glycoprotein CD52 (54)	NK cells	Expansion of CD56^bright^ NK cells with no altered cytolytic function (54)
Dimethyl fumarate	Nrf2 activator with immunomodulating, anti-inflammatory and anti-oxidative effects (55)	NK cells	Expansion of CD56^bright^ NK cells (56) decrease in absolute numbers of CD56^dim^ cells (55)
Fingolimod	Sphingosine 1-phosphate receptor agonist (57)	NK cells ILCs 1-3	Decreased number of CD56^bright^ NK cells (55, 57, 58) CD56^dim^ NK cells are increased in the circulation (54, 58) Percentage of total NK cells increased (59) Interrupted ILC circulation in both humans and mice (3) -ILC2 migration blockade to lung or bone marrow in mice (60)

**has been withdrawn from the market in 2018.

IFN-β reduces T-cell proliferation, leads to a reduction of MHC class II molecule expression, and lowers IL-17A, TNF, and IFN-γ production in Th17/Th1 cells (47). It also upregulates MHC class I, which is the main inhibitory ligand for CD56^dim^ NK cells thereby dampening cytotoxicity of the CD56^dim^ NK cell population (61). In parallel, IFN-β alters the CD56^bright^ and dim population ratio in the peripheral blood with an expansion of CD56^bright^ NK cells after treatment (48). Whether these changes in the NK cell immune phenotype are relevant for therapeutic success is not understood (49, 61). In patients treated with Natalizumab- a humanized alpha 4 integrin (CD49d) antibody inhibiting leukocyte migration, an increase of total NK cells and CD56^bright^ NK cells in blood comes concomitantly with reduced NK cell numbers in the CSF suggesting that transmigration of NK cells into the CNS is CD49d dependent (50, 51, 53). Daclizumab - a monoclonal antibody blocking the IL2 receptor α (CD25) is assumed to reduce early T-cell activation by the expansion of CD56^bright^ NK cells (52, 62). Daclizumab both boosts NK cell cytolytic function in a DC-dependent manner and renders antigen-activated T cells more sensitive toward NK-mediated lysis, thus restoring defective NK cell-mediated control of T cell activity in MS (51, 52). Next to this, in MS patients treated with Daclizumab, a decrease in circulating ILCs can be found as well as a shift in the phenotype of ILCs, from LTis toward an NK cell lineage (44). However, Daclizumab has been withdrawn from the market in 2018 due to cases of severe inflammatory brain disease with fatal outcomes (63).

Alemtuzumab, a humanized monoclonal antibody that is specific for the membrane glycoprotein CD52, is highly effective in long-lasting suppression of the disease activity in RRMS patients (54). A study investigating the phenotype and effector function of innate immune cells in RRMS patients shows that the decrease of CD4^+^ T-cells was accompanied by an increase in ILCs which mainly was due to an expansion of the CD56^bright^ NK cells although it has to be mentioned that the cytolytic function of NK cells was not altered 6 months after alemtuzumab treatment (54).

DMF is an Nrf2 activator with immunomodulating, as well as anti-inflammatory and anti-oxidative effects (55). Studies show that MS patients treated with DMF have reduced percentages of pro-inflammatory CD4^+^ and CD8^+^ T cells and an expansion of CD56^bright^ NK cells and a modest increase in absolute numbers of CD56^dim^ cells (55, 56). FYT20, a sphingosine 1-phosphate receptor agonist used as an oral compound for the treatment of MS shows efficacy in reducing inflammation in the CNS of MS patients (57). NK cells express two receptors affected by this treatment- S1PR1 and S1PR5 consequently affecting their egress from the lymph nodes (3). Data on the effect of long-term FTY20 treatment on NK cells are however contradictory (57, 59, 64). In a clinical phase IV trial in 17 RRMS patients, the longitude impact of FYT20 treatment on NK cells was assessed (58). An increased frequency of circulating CD56^dim^ mature NK cells was found while the frequency of CD56^bright^ and CD127^+^ ILCs decreased over time (58). This is in line with two other studies reporting a significant decrease of CD56^bright^ NK cells in the peripheral blood of FYT20 treated patients (55, 57). Other studies found that in FYT20 treated MS patients the percentage of NK cells increased when compared to treatment-naïve patients (56) or that NK cells were not affected by FYT20 (59).

A study investigating whether ILC subsets such as ILC1-3, like NK cells, also express S1PR1, which is targeted by FYT20 found that ILCs 1-3 indeed express S1PR1 in both humans and mice (65). A comparison between treatment-free MS patients and patients receiving FYT20 further showed that the absolute number of total ILC from all ILC subsets was reduced in the peripheral blood. Similar results were also found in mice (20). In mice, injections with FYT20 resulted also in a blocked ILC2 migration from intestines to lung or bone marrow during an inflammatory state (65) indicating that FYT20 treatment affects several immune subsets.

## Conclusion and Future Perspectives

NK cells and other ILCs have long been neglected as players in MS but accumulating evidence shows that ILCs indeed play a role in the disease pathology. While on the one hand ILCs have been described to prevent autoimmunity, several studies implicated them in a disease-promoting role in EAE by influencing T-cells and releasing cytokines that enhance the pro-inflammatory response.

In addition*, in vitro*, and *in vivo* mouse studies suggest that NK cells are also able to directly target oligodendrocytes- adding a layer of complexity as they might not only be involved in the neuroinflammation aspect but also directly in the neurodegenerative portion of MS.

Different depletion methods of NK cells and additional different experimental conditions such as gender and age of mice, peptides used for EAE induction, or housing conditions (SPF status, microbiota) might play a role in the contradictory findings of the aforementioned studies. As well the amount of peptides used and whether a direct EAE induction or passive EAE induction was used, could additionally have influenced the outcome of the studies. Next to this, several studies have been performed before the discovery or strict classification of different ILC subsets making it hard to clearly interpret their role in MS. In addition, a certain plasticity of ILCs has been reported and, likely, ILCs can also adapt to environmental cues during neuroinflammation changing their transcription factor and cytokine profile.

Given that ILCs interact with other immune cells such as T- and B-cells, it would be important to get a deeper understanding of the interaction of the different innate and adaptive immune subsets during the disease initiation and progression. The fact that all types of ILCs are recruited to the meninges and CNS indicates a complex interaction of these cell types. Another question that still needs to be addressed is whether ILC subsets are only involved in the initiation phase of disease by opening the blood-brain barrier and recruiting other immune cells, thereby triggering local T-cell and B-cell responses, or whether they are also crucial for the maintenance and progression of the disease. Deepening our understanding of the role of ILCs in the progression and not the initiation phase would be of importance to design better therapies.

Although there are several treatment options for MS, most of the treatments are perceived as burdening the patients due to the administration route and severe side effects Consequently, there is a high unmet medical need for new therapeutics which are more patient-friendly and possibly delay and minimize the neurodegenerative part of the disease (20). Due to their pharmacological safety, tolerability, and efficacy profiles, peptides present a unique starting point for creating new treatments for a wide range of diseases (58, 60). Thus, it is attractive to further explore the option of peptide therapeutics for MS. Indeed, the circular plant peptide T20J kB1 (T20K) has been shown to silence T-cell proliferation in an IL-2-dependent mechanism, and treating MOG-induced mice with the peptide led to a significantly delayed onset of clinical symptoms in the EAE model (58). This compound is currently tested in clinical trials and it is attractive to speculate that T20K might have an additional impact on innate lymphoid cells as IL-2 can promote activation and cell growth of ILCs.

Next to this, the finding of ILC subset residing in the meninges could be of deep interest since they lie outside of the blood brain barrier and are more targetable. It is therefore essential to further investigate how ILCs accumulate in the meninges. It is not clear yet whether meningeal ILCs are homeostatic gatekeepers and have the potential to locally proliferate in a disease-promoting environment or whether circulating ILCs are recruited to the CNS during the disease.

In conlusion, increasing our understanding of the complex interplay of different immune cells including ILCs during the disease initiation and progression will be crucial to find alternative treatment options.

## Author Contributions

NH, DG, SM, and BB discussed the literature and wrote the manuscript. NH generated all figures. All authors contributed to the article and approved the submitted version.

## Funding

NH and DG are supported by a grant from the Austrian Science Fund (FWF): ZK-81B. SM is supported by the JESH program of the Austrian Academy of Sciences. BB is supported by the Fonds voor Wetenschappelijk Onderzoek (FWO), the Belgian Charcot Foundation, Stichting MS Research, Multiple Sclerosis International Federation (MSIF) and MoveS.

## Conflict of Interest

The authors declare that the research was conducted in the absence of any commercial or financial relationships that could be construed as a potential conflict of interest.

## Publisher’s Note

All claims expressed in this article are solely those of the authors and do not necessarily represent those of their affiliated organizations, or those of the publisher, the editors and the reviewers. Any product that may be evaluated in this article, or claim that may be made by its manufacturer, is not guaranteed or endorsed by the publisher.
